# Phototherapy 660 nm for the prevention of radiodermatitis in breast cancer patients receiving radiation therapy: study protocol for a randomized controlled trial

**DOI:** 10.1186/1745-6215-15-330

**Published:** 2014-08-20

**Authors:** Marina Moreira Costa, Sidney Benedito Silva, Ana Luiza Pereira Quinto, Priscilla Furtado Souza Pasquinelli, Vanessa de Queiroz dos Santos, Gabriela de Cássia Santos, Daniela Francescato Veiga

**Affiliations:** Department of Oncology, Hospital do Cancer de Barretos, Rua Antenor Duarte Vilela, 1331, Barretos, SP CEP 14784-400 Brazil; Department of Physiotherapy, Universidade do Vale do Sapucaí, Avenida Alfredo Custódio de Paula, 320, Pouso Alegre, MG CEP 37550-000 Brazil; Department of Radiotherapy, Hospital do Cancer de Barretos, Rua Antenor Duarte Vilela, 1331, Barretos, SP CEP 14784-400 Brazil; Department of Radiotherapy, Oncominas, Rua Benedito Valdetário e Silva, 80, Pouso Alegre, MG CEP 37550 Brazil; Department of Plastic Surgery, Universidade do Vale do Sapucaí, Avenida Prefeito Tuany Toledo, Pouso Alegre, MG CEP 37550-000 Brazil; Department of Plastic Surgery, Universidade Federal de São Paulo, Rua Napoleão de Barros, 715, São Paulo, CEP 04024-002 Brazil

**Keywords:** Breast neoplasms, Laser therapy, Low-level, Radiodermatitis, Prevention & control

## Abstract

**Background:**

Breast neoplasms are the second most common type of cancer worldwide, and radiation therapy is a key component of their treatment. Acute skin reactions are one of the most common side effects of radiation therapy, and prevention of this adverse event has been investigated in several studies. However, a clinically applicable, preventative treatment remains unavailable. It has been demonstrated that application of a low-power laser can promote tissue repair. Therefore, the aim of this trial is to evaluate the effectiveness of an indium gallium aluminum phosphorus (InGaAIP) laser operated at 660 nm in preventing radiodermatitis in women undergoing adjuvant radiotherapy for breast cancer.

**Methods/Design:**

This is a two-arm, randomized controlled trial. A total of 52 patients undergoing radiotherapy for breast cancer (stages I to III) will be enrolled. Patients will be randomly assigned to an intervention group to receive laser therapy (n = 26) or a control group to receive a placebo (n = 26). The laser or placebo will be applied five days a week, immediately before each radiotherapy session. Skin reactions will then be graded weekly by a nurse, a radiotherapist, and an oncologist (all of whom will be blinded) using the Common Toxicity Criteria (CTC) developed by the National Cancer Institute and the Acute Radiation Morbidity Scoring Criteria developed by the Radiation Therapy Oncology Group. Patients will also answer a modified visual analogue scale for pain (a self-evaluation questionnaire). Primary and secondary outcomes will be the prevention of radiodermatitis and pain secondary to radiodermatitis, respectively.

**Discussion:**

The ideal tool for preventing radiodermatitis is an agent that mediates DNA repair or promotes cell proliferation. Application of a low-power laser has been shown to promote tissue repair by reducing inflammation and inducing collagen synthesis. Moreover, this treatment approach has not been associated with adverse events and is cost-effective. Thus, the results of this ongoing trial may establish whether use of a low-power laser represents an ideal treatment option for the prevention of radiodermatitis.

**Trial registration:**

ClinicalTrials.gov identifier: NCT02003599. Registered on 2 December 2013.

**Electronic supplementary material:**

The online version of this article (doi:10.1186/1745-6215-15-330) contains supplementary material, which is available to authorized users.

## Background

Approximately 90% of patients with breast cancer who are treated with adjuvant radiotherapy develop acute skin reactions [1]. Interventions to reduce this adverse side effect are needed since radiodermatitis may impact a patient’s adherence to treatment, and, with increased severity, can lead to the discontinuation of a potentially curative treatment [1, 2].

Acute radiodermatitis is a reaction caused by secondary skin exposure to ionizing radiation. Ionizing radiation impairs the ability of cells to proliferate quickly, and this leads to a skin reaction. This complex process involves DNA damage and changes in proteins, lipids, and carbohydrates. At the tissue level, this injury to the skin includes basal cell destruction and depletion of skin cells. As the migration of basal cells to the skin surface is compromised by radiation, flaking of the skin develops [1, 3]. Initially manifesting as erythema between the first and fourth weeks of radiation treatment, this condition can progress to ulceration up to three months after treatment [4, 5].

Known risk factors for radiodermatitis include total radiation dose, fractionation scheme, type of equipment used, volume of tissue irradiated (such as breast volume), and the radiosensitivity of the tissues involved. It is believed that patient-related factors, such as smoking habit, chronic diseases (including diabetes mellitus), and concomitant anticancer treatment can also interfere with skin reactions by affecting the healing process [6].

Prevention of this adverse event has been investigated in several studies [3, 7–9]. However, there is currently no clinically applicable prophylaxis available. As suggested by the Cancer Care Ontario Guidelines, the efficacy of topical agents remains controversial as well, and there is not sufficient evidence to indicate a prophylactic regimen using these agents for radiodermatitis.

Bolderston *et al*. [7] conducted a systematic review on the prevention and management of acute skin reactions related to radiation therapy. Unfortunately, the trials included in this systematic review were highly heterogeneous (the trials evaluated different kinds of treatments such as topical steroid creams, washing practices, sucralfate, Biafine cream, oral enzymes, amifostine, topical acid cream, aloe vera, chamomile cream, and dressings), and they concluded that there was insufficient evidence to support or refute specific topical or oral agents for the prevention or management of acute skin reactions [7].

Although various topical agents have been used to treat acute skin reactions, there is not sufficient evidence to establish a standardized recommendation for their application [3, 7]. In 2012, Zhang *et al*. [3] performed a meta-analysis on the use of topical agent therapy for the prevention and treatment of radiodermatitis. They concluded that topical agents could not prevent or treat radiodermatitis, probably because the topical agents chosen did not address the pathophysiology of radiodermatitis. An ideal agent for the prevention of radiodermatitis would mediate the repair of DNA damage or promote cell proliferation [3].

It was demonstrated that the cumulative effect of a low-power laser, when used at the appropriate dose, promoted tissue repair, possibly due to reduced inflammation and to the induction of collagen synthesis [10]. Specifically, laser treatment had a positive effect on injured fibroblasts by directly stimulating their growth, by affecting the expression of genes related to cell migration, DNA repair, ion channels, membrane potential, and cellular metabolism. It is hypothesized that the sum of these events contribute to ulcer healing through an increase in collagen synthesis, microcirculation, and suppression of apoptosis [11].

The application of a low-power laser for the prevention of oral mucositis is already well-established [12–15]. Moreover, the application of laser treatments to patients with breast cancer has also been found to be safe [16], and this method currently represents an option for treatment of lymphedema, a side effect of the surgical treatment of breast cancer [17–20]. Furthermore, a meta-analysis demonstrated that there are no side effects due to use of a laser during these treatments [14].

Previously, only one study has investigated the use of photomodulation by using light emitting diodes (LEDs) [21]. In this study, breast cancer patients were treated with radiation therapy following a lumpectomy, and LED photomodulation treatments were administered immediately following intensity-modulated radiation therapy (IMRT). These photomodulation treatments were found to reduce the incidence of skin reactions having a National Cancer Institute (NCI) grading of 1, 2, or 3 [21].

When laser therapy was applied to oral mucositis secondary to radiotherapy and chemotherapy, the incidence of grade 3 mucositis was reduced from 35.22 to 7.62% [22]. Based on these results, it is hypothesized that laser treatments may also represent a prophylaxis for radiodermatitis, particularly since there is currently no effective treatment for this side effect of radiotherapy. Therefore, this randomized controlled trial was designed to assess the effectiveness of laser therapy for the prevention of radiodermatitis.

## Methods/Design

### Study aims

The primary aim is to evaluate whether an indium gallium aluminum phosphorus (InGaAIP) laser operated at 660 nm can minimize the occurrence of radiodermatitis in breast cancer patients undergoing adjuvant radiotherapy. The secondary aim is to evaluate the ability of these laser treatments to decrease the pain associated with radiodermatitis.

#### Ethical issues

Ethical Committees of the Universidade do Vale do Sapucaí and the Hospital do Cancer de Barretos have approved this study protocol (approval numbers 564.777 and 626.876 respectively). Only participants who agree to provide written informed consent will be enrolled.

#### Study design and setting

This is a two-arm, parallel group, randomized controlled trial that will be conducted at the Hospital do Cancer de Barretos (Brazil), which treats annually approximately 600 patients who undergo adjuvant radiotherapy. Patients will be recruited from the radiotherapy department of this hospital.

#### Sample size

This study is powered to detect a reduction in the rate of radiodermatitis grade 3. To achieve an acceptable type 1 error of 5% and a type 2 error of 20%, the required sample size is 26 patients per arm (one-tailed test). We were not able to find data from breast cancer patients to use in the sample size calculation, thus it was performed based on data from the study of Bensadoun *et al*., which used a low-energy He/Ne laser to prevent radiation-induced mucositis in head and neck cancer patients [22]. These authors found not only a reduction in mucositis grade 3 (35.22% occurrence in control group versus 7.62% in laser group), but also a reduction in oral pain as assessed by patients, due to the preventive use of laser application.

#### Inclusion criteria

Female patients older than 18 years of age with a histological diagnosis of breast cancer (stages I to III) who underwent breast-conserving surgery or mastectomy without breast reconstruction with proposed adjuvant conventional radiotherapy according to the clinical protocol of Hospital do Cancer de Barretos (at full volume: ‘hot spot’ ≤115%, according to the International Commission on Radiation Units and Measurements (ICRU)) will be enrolled in this trial.

All patients will undergo radiotherapy according to the radiotherapy protocol of Hospital do Cancer de Barretos. The maximal point dose will not exceed 115% of the prescription whole breast dose (for example, the maximal point dose will not exceed 57.5 Gy for a prescribed dose of 50 Gy).

#### Exclusion criteria

Patients who have undergone a mastectomy with immediate breast reconstruction and those suffering from collagen diseases will be excluded from this study. Patients who do not meet the criteria for planning radiotherapy will also be excluded (patients with maximal point dose >115%).

#### Group assignment

All patients undergoing radiotherapy for breast cancer will be assessed for eligibility criteria (including the dose of radiation) immediately after the radiotherapy planning. Eligible patients will be informed about the study and invited to participate. A total of 52 patients undergoing radiotherapy for breast cancer (stages I to III) will be prospectively enrolled after providing informed consent.

The participants will be allocated to an intervention group (laser therapy, n = 26) or a to a control group (placebo, n = 26). The latter will receive treatment from a deactivated laser unit that does not deliver radiation.

Group allocation will be determined using randomly generated computer sequences (Software R™, R Foundation for Statistical Computing, Vienna, Austria) [22]. The allocation concealment will be achieved by using a sealed opaque envelope with patient’s number in the study, which will be opened by the physiotherapist responsible for the intervention immediately before the first laser or placebo application.

The radiotherapist, the oncologist, the nurse, and the patients will be blinded to the allocation outcome. Only those administering the intervention (physiotherapist) will know the allocation of patients into the groups.

#### Baseline procedures and interventions

Patients will undergo radiotherapy according to the radiotherapy protocol of Hospital do Cancer de Barretos. Protocols vary from 25 to 30 sessions of radiotherapy, taking into account the need of sequential tumor bed boost in some patients.

**Figure 1:**
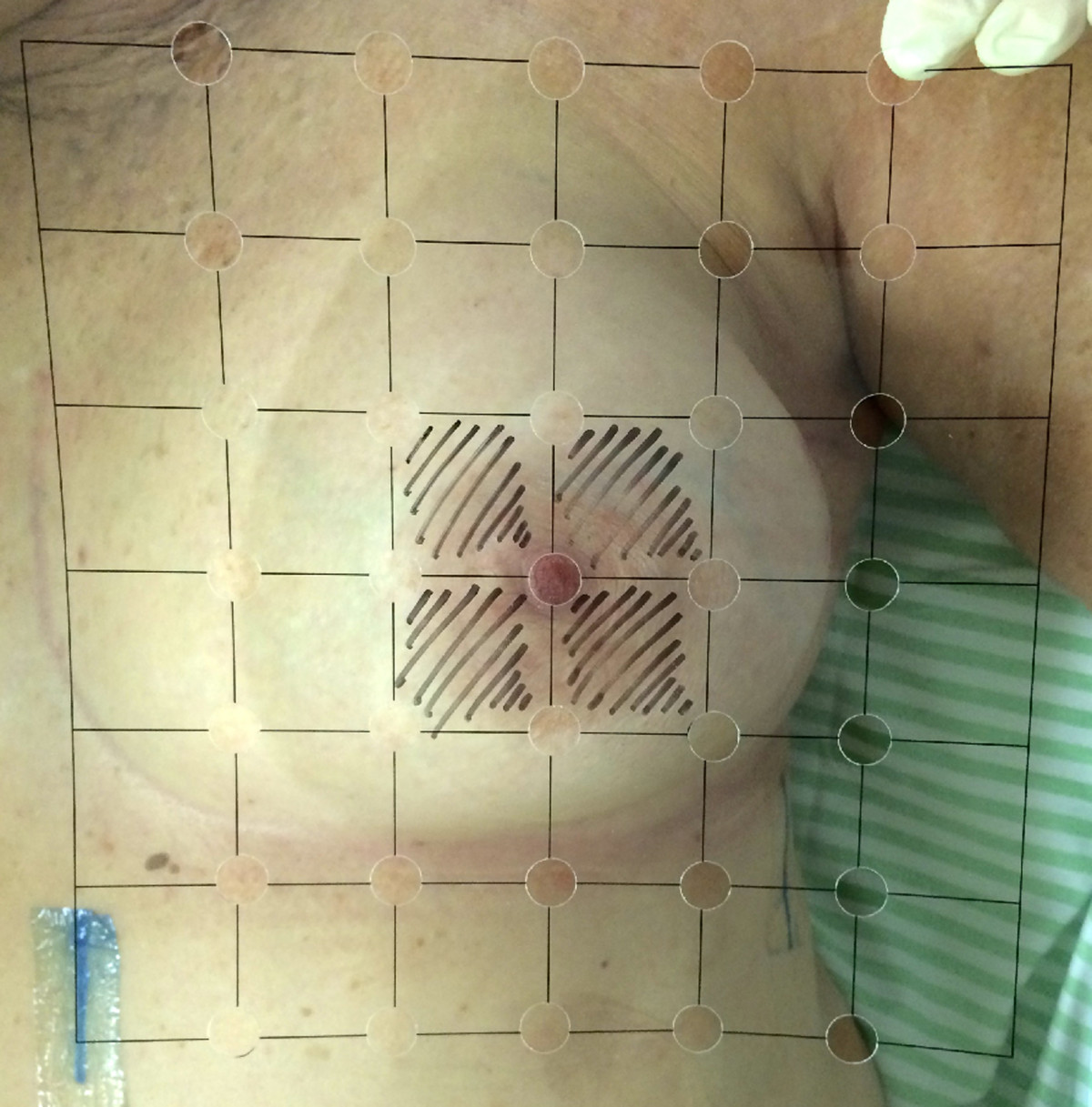
**Plastic guide with a grid of 35 points to guide the application of the laser or placebo.**

A pilot study was performed to adjust laser application methods, since there is no data about this in literature. For both intervention and control groups, a center point will be marked within an 18 × 18 cm [2] size area. Then, a plastic guide with a grid of 35 points will be centered over the surgical bed to guide the application of the laser or placebo (Figure 1). Five days a week, patients in intervention group will receive a laser treatment that is applied for 22 minutes (38 seconds each point) within 12 hours prior to each radiotherapy session. For the control group, the same procedure will be performed without activation of the laser. However, the appearance and noise of this placebo will be identical to the treatment group. In addition, both groups of patients will be blindfolded and will wear protective goggles during the treatments.

The center of the diagram is the meeting center of radiotherapy fields. In patients where this is not possible (patients radiating supraclavicular fossa), the nipple will be used as reference point for the center; and, in mastectomy patients, the midpoint between the tattoos will be used (as routine for radiation therapy, the patients undergoing radiation therapy in the hospital are routinely tattooed in two points, mid-axillary line and sternal; its midpoint roughly corresponds to nipple’s localization).

Patients allocated to the intervention group will receive laser treatments using a Photon Laser III (DMC™, São Carlos - SP, Brazil), approved by ANVISA (Agência Nacional de Vigilância Sanitária) for medicinal purposes. This InGaAIP laser emits a pulsed 660 nm beam with contact, with an average output of 80 mW. The laser energy is 3 J, with a power density of 80 mW and a dose emission of 108 J/cm [2]. The distribution of the light beam in the marking will be on points with a radius of 1 cm. Clinical and demographic data will be collected before the first radiotherapy session, including schemes of chemotherapy, breast volume, smoking habit, chronic diseases, and concomitant anticancer treatment, as they are factors that can interfere with the development of radiodermatitis.

After the second week of radiotherapy, patients will be evaluated for radiodermatitis weekly, after the laser or placebo application. They will also complete a modified visual analogue scale for pain (a patient self-evaluation questionnaire, validated for use in Brazil) [23]. Skin reactions and pain will be continue to be graded weekly until the end of treatment, and this evaluation will be independently performed by three previously trained evaluators: a nurse, a radiation oncologist, and an oncologist. The Common Toxicity Criteria (CTC 4.0), developed by the NCI [24], and the Acute Radiation Morbidity Scoring Criteria developed by the Radiation Therapy Oncology Group (RTOG) [5] will be used as guidelines.

The CTC was first described in 1982 and the version we will is the 4.0. It classifies radiodermatitis in degrees: 1 (faint erythema or dry desquamation), 2 (moderate to brisk erythema; patch moist desquamation, mostly confined to skin folds and creases; moderate edema), 3 (moist desquamation in areas other than skin folds and creases; bleeding induced by minor trauma or abrasion), 4 (life-threatening consequences; skin necrosis or ulceration of full thickness dermis; spontaneous bleeding from involved site; skin graft indicated) and 5 (death) [24].

Also in 1982, The RTOG developed the Criterion score for Acute Radiation Morbidity - Acute Radiation Morbidity Scoring Criteria, to classify the effects of radiotherapy. This criterion identifies grades 0 (no change over baseline), 1 (follicular faint or dull erythema, epilation, dry desquamation, decreased sweating), 2 (tender or bright erythema, patchy moist desquamations, moderate edema), 3 (confluent, moist desquamation other than skin folds, pitting edema) and 4 (ulceration, hemorrhage, necrosis) [5].

The visual analogue scale for pain was adapted and validated for use in Brazil by Ferraz *et al*. [23], in 1990. It is a patient self-evaluation scale, ranging from 0 to 10, with 0 meaning no pain and 10 meaning maximum pain [23].

Grading of radiodermatitis and pain assessment and will be repeated 90 days after the beginning of radiotherapy. If any of the evaluators perceive any adverse event that could be attributed to the intervention, or if the patient withdraws consent at any time, this patient will be immediately withdrawn from the study.

Both arms will follow the institutional skin care protocol, which consists of chamomile cream 10% with silicone cream 5.5%. This concoction will be used three times a day for the duration of the radiotherapy.

#### Outcome measures

The primary outcome is assessment and grading of radiodermatitis. The secondary outcome is pain assessment.

#### Endpoints

Participation is in this study is considered complete 90 days after the beginning of radiotherapy. However, exit points include a delay in radiotherapy greater than 10 days (if the break is not caused by radiodermatitis), or non-attendance of the patient at weekly review sessions.

#### Statistical analysis

Statistical analyses will be performed using Statistical Package for Social Sciences (SPSS) software v.18 (SPSS Inc., Chicago, Illinois, United States) and Software R™, (R Foundation for Statistical Computing, Vienna, Austria) [25]. The rejection level for the null hypothesis will be fixed at 5% (α ≤0.05). The pondered kappa coefficient will be used to compare responses among evaluators. The McNemar test will be used to compare levels of pain between groups (one test will be performed weekly). For inter-group assessments of the proportion of radiodermatitis, the Chi-square test will be used to compare group intervention and control.

## Discussion

Radiodermatitis is one of the most common side effects of radiation therapy, and despite advances in treatment techniques, skin reactions remain inevitable [1, 26]. Moreover, this adverse event can represent a significant source of pain or discomfort and can lead to limited daily activities, a decrease in quality of life, decreased patient adherence to treatment, or even an interruption of treatment [1]. Consequently, it is important that efforts be made to prevent radiodermatitis.

Low-power laser treatments have been well-established for the prevention and treatment of oral mucositis and for the treatment of lymphedema [12–15, 17–20]. Moreover, according to a recent meta-analysis on radiation-induced mucositis, low-level laser therapy at a dose of 2 to 3 J/cm [2] that was administered in daily applications, or at least three times a week, did not result in any adverse effects [14].

Regarding lymphedema, a recent systematic review presented moderate to strong evidence that low-level laser treatments are effective for the management of this condition [17]. For the 230 patients analyzed, a dose of 1 to 2 J/cm [2] per point applied, with several points covering the fibrotic area, reduced limb volume [17]. Furthermore, Carati *et al*. [20] conducted a prospective, double-blind, randomized controlled study with 61 patients who underwent mastectomy and then developed lymphedema. There were 28 patients in the placebo group and 33 patients in the treatment group. For the latter, a 904 nm RianCorp LTU 904H laser was applied to 17 points at 2 cm intervals (using a plastic guide) in the region of the axilla for each patient. For the placebo group, the laser unit was deactivated and no radiation was delivered. Moreover, this unit was indistinguishable from the active unit. The total energy applied was 1.5 J/cm [2], and this treatment was found to reduce the volume of arm lymphedema in 31% of the patients that completed three months of this treatment [20].

Despite all the training conducted for the grading of radiodermatitis, the CTC and RTOG’s scales are dependent on observers, and this may lead to a grading bias. Unfortunately we cannot completely eliminate subjectivity in evaluating the radiodermatitis grade.

In the absence of clinical guidelines for the prevention of radiodermatitis, treatment of this side effect of radiation remains a challenge. However, based on the effectiveness of laser treatments for the prevention of oral mucositis, a clinical trial to investigate the beneficial application of laser treatments as a prophylaxis for radiodermatitis was established. It is possible that the results of this trial may provide a standard treatment for the prevention of acute skin reactions that develop following radiotherapy.

### Trial status

Recruitment began on April 2014, and it is ongoing. A total of 26 patients were enrolled by the end of June 2014.

## Authors’ original submitted files for images

Below are the links to the authors’ original submitted files for images. Authors’ original file for figure 1

## Abbreviations

ANVISA: Agência Nacional de Vigilância Sanitária
CTC: Common toxicity criteria
Gy: Grays (dose administrada de radioterapia)
ICRU: International commission on radiation units and measurements
IMRT: Intensity-modulated radiation therapy
InGaAlP: Indium gallium aluminum phosphorus
LED: Light emitting diode
NCI: National cancer institute
RTOG: Radiation therapy oncology group.
