# Supplementary figures and images for: Phototherapy 660 nm for the prevention of radiodermatitis in breast cancer patients receiving radiation therapy: study protocol for a randomized controlled trial
Source: Trials. 2014 Aug 20;15:330. doi: 10.1186/1745-6215-15-330 (PMC4148541; doi:10.1186/1745-6215-15-330)

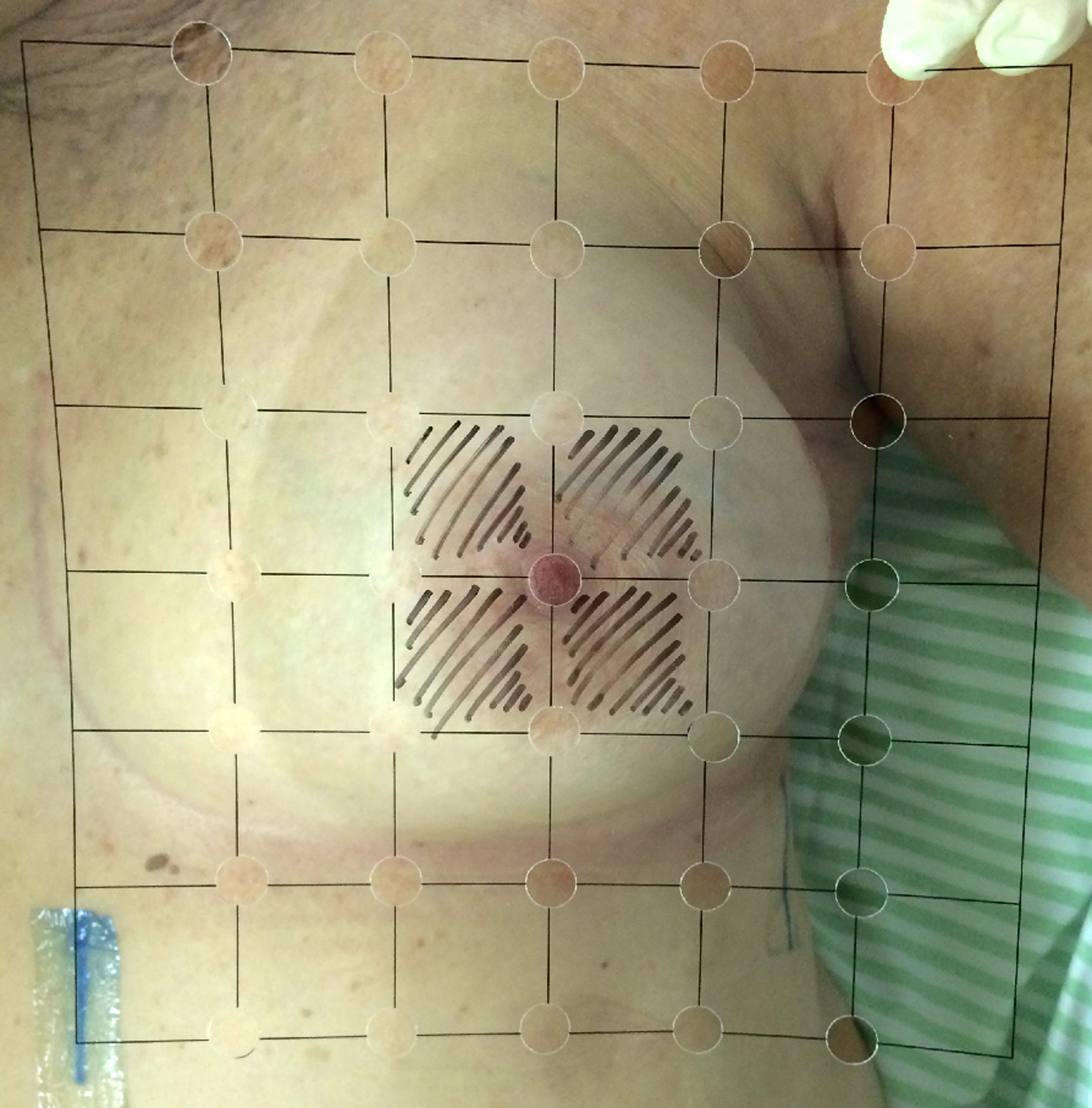

Supplement: Supplementary file 1 — Authors’ original file for figure 1 [file 13063_2014_2203_MOESM1_ESM.tif]
